# Prevalence of Thyroid Autoimmunity in Women with Recurrent Pregnancy Loss

**DOI:** 10.3390/medicina57020096

**Published:** 2021-01-22

**Authors:** Myrna Souraye Godines-Enriquez, Silvia Miranda-Velásquez, María Magdalena Enríquez-Pérez, Lidia Arce-Sánchez, Nayeli Martínez-Cruz, Claudia Montserrat Flores-Robles, Patricia Aguayo-González, Fela Vanessa Morales-Hernández, Alma Villarreal-Barranca, Blanca Vianey Suárez-Rico, Araceli Montoya-Estrada, José Romo-Yáñez, Enrique Reyes-Muñoz

**Affiliations:** 1 Education in Health Sciences, National Institute of Perinatology, Ministry of Health, Mexico City 11000, Mexico; dra.myrnagodines@gmail.com; 2 Reproductive Gynecology Department, National Institute of Perinatology, Ministry of Health, Mexico City 11000, Mexico; dra_silvia_miranda@hotmail.com (S.M.-V.); pagonzalez2108@yahoo.com.mx (P.A.-G.); vanesamoh@gmail.com (F.V.M.-H.); 3 Department of Obstetrics, National Institute of Perinatology, Ministry of Health, Mexico City 11000, Mexico; magdishon@yahoo.com; 4 Department of Endocrinology, National Institute of Perinatology, Ministry of Health, Mexico City 11000, Mexico; li_arce@yahoo.com.mx (L.A.-S.); nayemc_21@hotmail.com (N.M.-C.); montseflores@hotmail.com (C.M.F.-R.); 5 Coordination of Education and Research, Hospital de la Mujer, Ministry of Health, Mexico City 11340, Mexico; alma.vvb@gmail.com; 6 Direction of Research, National Institute of Perinatology, Ministry of Health, Mexico City 11000, Mexico; blancasuarezrico@gmail.com; 7 Coordination of Gynecological and Perinatal Endocrinology, National Institute of Perinatology, Ministry of Health, Mexico City 11000, Mexico; ara_mones@hotmail.com (A.M.-E.); jryz@yahoo.com (J.R.-Y.)

**Keywords:** thyroid autoimmunity, recurrent pregnancy loss, antithyroperoxidase antibodies, antithyroglobulin antibodies

## Abstract

*Background and objectives:* Thyroid autoimmunity (TAI) has been associated with a significantly increased risk of miscarriage in women with recurrent pregnancy loss (RPL). The aim of this study was to determine the prevalence of TAI in women with RPL and compare the clinical characteristics of positive and negative TAI women. *Materials and Methods:* This is a retrospective cross-sectional study; 203 women with RPL were included. Thyroid profile, anti-thyroid peroxidase (TPO-Ab), and anti-thyroglobulin (TG-Ab) antibodies were measured in all participants. Clinical characteristics and causes of RPL were compared between positive and negative TAI. *Results:* Prevalence of TAI was 14.8%; prevalence of positive TPO-Ab and TG-Ab was 12.3% and 4.9%, respectively. Women with TAI had significantly higher concentrations of thyrotropin (TSH) compared to women without TAI (4.8 ± 3.8 versus 3.1 ± 1.1, *p* = 0.001). There was no significant difference in age, the number of gestations, miscarriages, state of antiphospholipid antibodies (aPL), or causes of RPL between women that were TAI-positive versus TAI-negative. Prevalence of positive TAI by cause of RPL was: endocrine 7/25 (28%), genetic 1/5 (20%), autoimmune 1/5 (20%), anatomic 8/55 (14.5%), and unexplained cause 13/112 (11.6%). *Conclusions:* The prevalence of TAI in women with RPL is 14.8%. Women with an endocrine cause have the highest prevalence of TAI.

## 1. Introduction

Recurrent pregnancy loss (RPL) is defined by two or more consecutive pregnancy losses [1,2] and is experienced by 1–3% of women of reproductive age [3]. The accepted etiologies for RPL are: chromosomal abnormalities, endocrine disorders such as hypothyroidism or uncontrolled diabetes mellitus, uterine anatomical anomalies, and antiphospholipid syndrome (APS). Other probable etiologies incorporate additional endocrine disorders, hereditary and/or acquired thrombophilias, immunological alterations, and environmental factors [4].

It has been described that 50% of RPL cases have an unexplained etiology [5]. In this context, it is hypothesized that an inadequate immunological interaction between mother and the embryo is the main factor related to adequate placental development, embryo survival, and maintenance of early pregnancy [6]. An altered maternal–fetal immunity may be responsible for serious gestational complications including RPL.

During the last decade, several observational studies have suggested that the presence of thyroid autoimmunity (TAI) is related to a significant increase in the risk of miscarriage [7,8]. Some of these studies have shown that serum thyrotropin (TSH) levels among women with a history of recurrent miscarriage and TAI are within normal parameters, but higher than those without TAI [9]. Additionally, two systematic reviews reported that in women who had a miscarriage, the average age of women with TAI was slightly higher than without TAI [9,10]. 

Although the mechanism involved is not clear, three possible explanations for the association of TAI with miscarriage have been proposed: (1) Pregnancy loss is an epiphenomenon and not a direct effect of TAI—the presence of TAI reflects a generalized activation of the immune system and specifically represents an increased reactivity against the fetus–placental unit [11]; (2) The presence of TAI may act as a factor of infertility and may delay conception; therefore, when women with TAI become pregnant, they have an increased risk of miscarriage due to advanced age [12,13,14]; (3) The loss of pregnancy may be secondary to a deficiency in thyroid hormone levels or a lower ability of the thyroid to adapt appropriately to pregnancy demands [15,16].

The treatment of euthyroid women with RPL and positive thyroid peroxidase antibodies (TPO-Ab) remains controversial. The empirical treatment with levothyroxine has been recommended by some researchers; however, it is not universally accepted, since the benefit of treatment has not been demonstrated in high-quality clinical trials [8]. The usefulness of screening and therapeutic intervention in women with RPL and TAI to improve the clinical outcome of future pregnancies is still uncertain [15]. A recent systematic review states that thyroid antibody screening in RLP is not supported by the actual published studies, and further randomized studies are needed to know if this practice should be recommended [8].

The prevalence of TAI in Mexican women with RPL is missing in the literature. The objective of this study was to determine the prevalence of TAI in women with PRL and compare the clinical characteristics of positive and negative TAI women. 

## 2. Material and Methods

A retrospective cross-sectional study was carried out at the RPL clinic of the Instituto Nacional de Perinatología between January 2013 and June 2014. The study was performed according to the principles of the Declaration of Helsinki and it was approved by the Ethics and Research Committees of the Instituto Nacional de Perinatología on 6th July 2015, with register number 212250-2102-10209-01-15. Data of participants were obtained from clinical records. 

### 2.1. Subjects and Procedures

Non-pregnant women with RPL according to 2018 European Society of Human Reproduction and Embryology (ESHRE) guidelines were included [17]. Women with a history of ectopic, molar pregnancies an uncomplete clinical record were excluded. All women were studied according with the 2011 Royal College of Obstetricians and Gynaecologists (RCOG) guidelines [18]. That included the study of the following factors: (a) Genetic factor: karyotype from peripheral blood sample to the couple when there were losses in the first trimester or when data of chromosomal alteration were presented in fetuses or the descendants of the couple. (b) Anatomical factor: studied by ultrasound and sonohysterography (SHG) and/or hysterosalpingography (HSG). Hysteroscopy was performed in women with a preliminary diagnosis or suspicion of Müllerian malformation. Special cultures for Chlamydia trachomatis, Ureaplasma, and Mycoplasma were requested previously for the study of the anatomical factors (HSG, SHG). Cervical incompetence (CI) was diagnosed through the history of pregnancy loss plus the dilator test. (c) Endocrine factor: complete thyroid profile, fasting glucose, prolactin, ultrasound, and blood androgens were done to search for prediabetes, diabetes, thyroid disease, hyperprolactinemia, polycystic ovarian syndrome (PCOS), or other hyperandrogenic disorders. Subclinical hypothyroidism was defined as TSH >4 mUI/L with normal total triiodothyronine (TT3) and normal free thyroxine (FT4). Overt hypothyroidism was defined as TSH value >10 mUI/L regardless TT3 or FT4 concentrations. Diabetes and prediabetes were defined according to the current criteria of the American Diabetes Association and PCOS according to the 2003 Rotterdam PCOS diagnostic criteria. Hyperprolactinemia was defined as serum prolactin higher than 25 ng/mL associated with oligo/amenorrhea or galactorrhea. (d) Immunological factors: anticardiolipin antibodies (IgM and IgG) and lupus anticoagulant in two tests with an interval of 12 weeks were done for the integration of APS. To integrate the diagnosis of APS titers over 40 U/mL for either lupus anticoagulant or anticardiolipin antibodies of immunoglobulin G and/or immunoglobulin M class has to be present. All women with a positive result for antiphospholipid antibodies were re-tested 12 weeks after the initial test. APS was considered in women with persistently positive tests. Hereditary thrombophilias including mutations for factor V Leiden and factor II (prothrombin), deficiency of protein C, S, and antithrombin were evaluated in women with a history of pregnancy loss during the second trimester.

A genetic cause of RPL was cataloged if abnormal karyotyping (chromosomal rearrangements) was detected in any parent; anatomic causes were defined if Müllerian malformations, intrauterine adhesions, submucosal fibroids, or endometrial polyps were discovered in imaging studies or if a diagnosis of cervical incompetence was made; autoimmune causes included APS and hereditary thrombophilias; endocrine causes included diabetes, overt hypothyroidism or hyperthyroidism, PCOS, or other hyperandrogenic disorders and hyperprolactinemia. These diagnoses were made by an attending physician in the RPL clinic based on the criteria previously described. 

No women were on levothyroxine (LT4) treatment at the time of performing basal thyroid profile and thyroid autoantibodies. According to institutional guidelines, all women with subclinical and overt hypothyroidism received LT4 therapy after the diagnosis.

### 2.2. Blood Samples

Whole blood 6 mL samples were obtained from an antecubital venipuncture in anticoagulated tubes with 2 mM ethylenediaminetetraacetic acid (BD Vacutainer, Franklin Lakes, NJ, USA) of each participant. The samples were centrifuged at 3000 rpm for 15 min to obtain the plasma and were stored in aliquots at −70 °C until their analysis.

### 2.3. Biomarkers

TSH, FT4, TT3, TPO-Ab, and Thyroglobulin (TG)-Ab were measured by chemiluminescence (IMMULITE 2000 Immunoassay System, Siemen’s Healthcare Diagnostics Inc., Deerfield, IL, USA). For TPO-Ab, the minimum detectable value was 10 IU/mL and the highest 1000 IU/mL; a value was considered positive if it was greater than 35 IU/mL. For TG-Abs, the calibration range was as high as 3000 IU/mL and the detection limit 20 IU/mL; values greater than 40 IU/mL were considered positive. Sensitivity for TSH is 0.002 mIU/L and the maximum value read is 75 mIU/L with an interassay variation coefficient of 10%; TSH normal range was considered 0.4–4.0 mIU/L. TT3 was analyzed with a detection range between 40 and 600 ng/dL and a sensitivity of 19 ng/dL. FT4 normal reference ranges are between 0.8 and 1.76 ng/dL, with a minimum detectable concentration of 0.22 ng/dL.

TAI was considered if any of TPO or TG-Abs were positive. Subclinical hypothyroidism was defined as TSH >4 mUI/L with normal total triiodothyronine (TT3) and normal free thyroxine (FT4). Hypothyroxinemia was determined when the FT4 value was less than 0.8 ng/dL and hyperthyroidism was determined when the FT4 was greater than 1.76 ng/dL.

Lupus anticoagulant (LA) was detected with HemosIL dRVVT Screen and HemosIL dRVVT X (Instrumentation Laboratory, Lexington, MA, USA). Russell’s viper venom method diluted in IL coagulation systems (dRVVT) was used; a value equal to or less than 47 s was negative. Both immunoglobulins (Ig) G and M of anticardiolipin antibodies (ACA) were analyzed using an assay based on the ELISA (Enzyme-Linked Immunosorbent Assay) technique for semi-qualitative detection, with a QUANTA Lite ACA IgM/IgG III assay (Inova Diagnostics, San Diego, CA, USA). A value of ACA IgG >10 units of phospholipids (PL) and >10 PL for ACA IgM were considered positive, negative results indicated the absence of anticardiolipin antibodies or levels lower than the cut-off point of the assay.

### 2.4. Sample Size

To find a TAI prevalence of 15% among women with RPL, with a 95% confidence level and a 5% error, the sample size required was 196 women. 

### 2.5. Statistical Analysis

The statistical analysis was performed using the Statistical Package for Social Science Software (SPSS 24, Chicago, IL, USA). Continuous variables were expressed as mean ± standard deviation; categorical variables were reported as the frequency and proportions. *T*-test was used to compare continuous variables between groups. The chi-square test or Fisher’s exact test were used to evaluate differences in proportions. Statistical significance was set at *p* ≤ 0.05.

## 3. Results

During the study period, 238 women with RPL were sequentially identified; 35 of them were excluded because they did not fulfill the inclusion criteria (3 due to a history of autoimmune hypothyroidism, 4 due to history of molar pregnancy, and 29 due to incomplete records). The remaining 203 women with RPL were included in the study. Prevalence of positive TAI was *n* = 30 (14.8%) and negative TAI was *n* = 173 (85.2%). Clinical characteristics of participants of the study at admission to the RPL clinic are shown in Table 1. There were no significant differences in age, height, weight, body mass index (BMI), number of gestations, miscarriages, FT4, TT3, and the frequency of aPL antibodies. The TSH concentrations were significantly higher in the TAI-positive group compared to the TAI-negative group (*p* = 0.0001). The prevalence of positive TAI was significantly higher in the group of women who received LT4 treatment 14/60 (23.3%) versus women who did not receive LT4 treatment 16/143 (11.2%), *p* = 0.02. 

The prevalence of positive TAI according to the cause of RLP was: genetic 1/5 (20%), anatomic 8/55 (14.5%), autoimmune 1/5 (20%), endocrine 7/25 (28%), and unexplained cause 13/112 (11.6%). There were no significant differences among causal factors between positive and negative TAI (Table 2). There was a higher prevalence of positive TAI in women with identified cause (56.7%) versus unexplained cause (43.3%); however, this was not significant (*p* = 0.22). 

In total, 76.6% of women with positive TAI had a BMI ≥ 25 kg/m^2^, and 60% were aged ≥30 years.

## 4. Discussion

The prevalence of positive TAI in women with RPL was 14.8%. Previous studies have reported that the prevalence of TAI ranged from 6.6 to 39% [19,20,21,22,23,24,25,26]. A possible explanation for the wide fluctuation may be the different test methods used to measure TAI. Some of these studies inform irregular results in external quality assessment systems despite being calibrated with the same reference material [27]. 

It is well recognized that TAI prevalence is affected by age. In one of the largest clinical trials [24], where 700 women with RPL and 200 healthy controls were included, the average age of patients with TAI in the RPL group was higher than the controls (33.3 years vs. 30.8 years; *p* < 0.01). Observing also that higher TAI titers increased as age in the RPL group in the range of 31 to 35 years, after which a decrease in frequency was observed. Two other meta-analyses also reported [9,10] that the mean age with positive TAI in women with RPL was slightly higher than women with negative TAI. In this study, there was no statistically significant difference in the mean age in women with positive and negative TAI. A significant difference was not found in the number of gestations or miscarriages in women with positive and negative TAI, which coincides with other works [11].

As in another research [27], women with unexplained RPL showed no significant difference in the prevalence of positive TAI compared to women with a known cause of RPL. The high prevalence of positive TAI in RPL women with an endocrine cause could be attributable to the inclusion of overt hypothyroidism women in this group. On the other hand, positive TAI could be the cause in the 11.6% of women labeled as having an unexplained cause.

In the present study, a higher concentration of TSH in women with positive TAI compared to negative TAI (4.8 mIU/L vs. 3.1 mIU/L) was observed, similar to the study by Yan et al. [27], which reported that women with positive TPO-Ab had a significantly higher TSH concentration than women with negative TPO-Ab. Moreover, this finding is in concordance with a meta-analysis, which reported a mean difference of 0.61 mIU/L in the TSH concentration among women with positive versus negative TAI [9].

It remains to be determined whether the detection and LT4 treatment in women with positive TAI and RPL improve the clinical outcome of future pregnancies. Vissenberg et al. [28], in a retrospective cohort of 202 euthyroid women with unexplained RPL, reported that positive TPO-Ab is associated with a lower live birth rate in euthyroid women with unexplained RPL and suggest that these women may benefit from treatment with levothyroxine. However, a more recent retrospective cohort study, with 1064 RPL women, found no difference in pregnancy outcomes based on TPOAb status in women with TSH between 2.5 and 4 mUI/L treated with LT4 [29], and a recent systematic review and meta-analysis found only one randomized controlled trial that evaluated LT4 treatment in euthyroid women with thyroid autoimmunity, while finding no benefit of levothyroxine treatment in this scenario [8].

The 2017 American Thyroid Association guidelines [30] state that insufficient evidence exists to conclusively determine whether LT4 therapy decreases pregnancy loss risk in TPOAb-positive euthyroid women who are newly pregnant, but suggests that women with a prior history of loss may be considered for low dose LT4 treatment (25–50 µg) given its potential benefits in comparison with its minimal risk. Concerning euthyroid women with autoantibody-positive who are attempting assisted reproductive technology (ART), ATA also states that insufficient evidence exists to make a recommendation for or against LT4 treatment, but mentions that a low dose can be considered. A 2017 meta-analysis failed to find an association between TAI and risk of miscarriage in women submitted to ART treatments [31].

It is important to remember that in those euthyroid women with positive antibodies who do not receive LT4 treatment during pregnancy, ATA recommends monitoring TSH every 4 weeks until midgestation and at least once near 30 weeks of gestation because of the risk of developing hypothyroidism [30]. Future prospective randomized clinical trials should focus on the potential health benefit of detection and treatment on pregnancy outcome in women with RPL and positive TAI.

## 5. Conclusions

The prevalence of TAI in women with RPL is 14.8%. Women with an endocrine cause had the highest prevalence of TAI. There are no significant differences in clinical characteristics between positive and negative TAI women. This study confirms a significant increase in TSH levels in women with RPL and positive TAI. 

## Figures and Tables

**Table 1:** Characteristics of 203 women with recurrent pregnancy loss at admission to the study.

Characteristics	Total *n* = 203	Positive TAI *n* = 30	Negative TAI *n* = 173	*p*
Age (years)	28.8 ± 4.6	28.9 ± 4.6	28.8 ± 4.7	0.87
Height (m)	1.55 ± 0.06	1.55 ± 0.05	1.55 ± 0.06	0.44
Weight (kg)	67 ± 12.6	65.8 ± 10.9	67.2 ± 12.9	0.56
BMI (kg/m^2^)	27.7 ± 4.8	27.5 ± 4.3	27.7 ± 4.9	0.79
Gestations	3.4 ± 0.9	3.7 ± 1.3	3.4 ± 0.9	0.11
Miscarriages	3.1 ± 0.8	3.2 ± 1.1	3.1 ± 0.7	0.23
TSH mUI/L	3.3 ± 2.3	4.8 ± 3.8	3.1 ± 1.1	0.0001
Free T4 mUI/L	1.1 ± 0.22	1.17 ± 0.25	1.14 ± 0.22	0.48
Total T3 mUI/L	126 ± 33	126.7 ± 39	126 ± 32	0.89
Positive lupic anticoagulant	13 (6.4)	3 (10)	10 (5.7)	0.64
Positive cardiolipin antibody IgM	16 (7.9)	2 (6.6)	14 (8)	0.92

Values expressed as mean and standard deviation or frequency and percentage (). BMI = Body mass index. TSH = Thyroid Stimulant Hormone, T4 = Thyroxine, T3 = Triiodothyronine. TAI: thyroid autoimmunity.

**Table 2:** Prevalence of positives TAI according to the causal factor.

Causal Factor	Total *n* = 203	Positive TAI *n* = 30	Negative TAI *n* = 173	*p*
Genetic	5 (2.5)	1 (3.3)	4 (2.3)	0.76
Anatomic	55 (27.1)	8 (26.7)	47 (27.2)	0.86
Autoimmune	6 (3)	1 (3.3)	5 (2.9)	0.65
Endocrine	25 (12.3)	7 (23.3)	18 (10.4)	0.09
Unexplained	112 (55.1)	13 (43.3)	99 (57.2)	0.22

Number in parentheses represents the percentage.

## Data Availability

Please contact the corresponding author for data requests.
